# Serum proteomics hint at an early T-cell response and modulation of SARS-CoV-2-related pathogenic pathways in COVID-19-ARDS treated with Ruxolitinib

**DOI:** 10.3389/fmed.2023.1176427

**Published:** 2023-05-24

**Authors:** Sara Völkel, Thomas S. Tarawneh, Laura Sacher, Aditya M. Bhagwat, Ihab Karim, Hildegard I. D. Mack, Thomas Wiesmann, Björn Beutel, Joachim Hoyer, Christian Keller, Harald Renz, Andreas Burchert, Andreas Neubauer, Johannes Graumann, Chrysanthi Skevaki, Elisabeth K. M. Mack

**Affiliations:** ^1^Institute of Laboratory Medicine, Philipps-University Marburg, Marburg, Germany; ^2^Department of Hematology, Oncology and Immunology, University Hospital Gießen and Marburg, Philipps-University Marburg, Marburg, Germany; ^3^Institute of Translational Proteomics, Philipps-University Marburg, Marburg, Germany; ^4^Institute for Biomedical Aging Research, Leopold-Franzens-Universität Innsbruck, Innsbruck, Austria; ^5^Department of Anesthesiology and Intensive Care Medicine, University Hospital Gießen and Marburg, Philipps-University Marburg, Marburg, Germany; ^6^Department of Anesthesiology, Intensive Care Medicine and Pain Therapy, Diakonie-Klinikum Schwäbisch Hall, Schwäbisch Hall, Germany; ^7^Department of Pulmonary and Critical Care Medicine, University Hospital Gießen and Marburg, Philipps-University Marburg, Marburg, Germany; ^8^German Center for Lung Research (DZL), Member of the Universities of Gießen and Marburg Lung Center, Gießen, Germany; ^9^Department of Nephrology, University Hospital Gießen and Marburg, Philipps-University Marburg, Marburg, Germany; ^10^Institute of Virology, Philipps-University Marburg, Marburg, Germany; ^11^Biomolecular Mass Spectrometry, Max Planck Institute for Heart and Lung Research, Bad Nauheim, Germany

**Keywords:** acute respiratory distress syndrome, COVID-19, proteomics, Ruxolitinib, SARS-CoV-2

## Abstract

**Background:**

Acute respiratory distress syndrome (ARDS) in corona virus disease 19 (COVID-19) is triggered by hyperinflammation, thus providing a rationale for immunosuppressive treatments. The Janus kinase inhibitor Ruxolitinib (Ruxo) has shown efficacy in severe and critical COVID-19. In this study, we hypothesized that Ruxo’s mode of action in this condition is reflected by changes in the peripheral blood proteome.

**Methods:**

This study included 11 COVID-19 patients, who were treated at our center’s Intensive Care Unit (ICU). All patients received standard-of-care treatment and *n* = 8 patients with ARDS received Ruxo in addition. Blood samples were collected before (day 0) and on days 1, 6, and 10 of Ruxo treatment or, respectively, ICU admission. Serum proteomes were analyzed by mass spectrometry (MS) and cytometric bead array.

**Results:**

Linear modeling of MS data yielded 27 significantly differentially regulated proteins on day 1, 69 on day 6 and 72 on day 10. Only five factors (IGLV10-54, PSMB1, PGLYRP1, APOA5, WARS1) were regulated both concordantly and significantly over time. Overrepresentation analysis revealed biological processes involving T-cells only on day 1, while a humoral immune response and complement activation were detected at day 6 and day 10. Pathway enrichment analysis identified the *NRF2-pathway* early under Ruxo treatment and *Network map of SARS-CoV-2 signaling* and *Statin inhibition of cholesterol production* at later time points.

**Conclusion:**

Our results indicate that the mechanism of action of Ruxo in COVID-19-ARDS can be related to both known effects of this drug as a modulator of T-cells and the SARS-CoV-2-infection.

## 1. Introduction

Severe acute respiratory syndrome corona virus 2 (SARS-CoV-2) was first described as the cause of severe pneumonia in Wuhan, China in December 2019 (1). The clinical presentation of corona virus disease 19 (COVID-19) is highly heterogenous ranging from asymptomatic courses to flu-like symptoms and all the way to lethal pneumonia with acute respiratory distress syndrome (ARDS) (2–4). Due to the rapid spread of the COVID-19 pandemic (5), treatment initially relied on repurposing of already available drugs (6) and standard-of-care management for ARDS including mechanical ventilation and other organ replacement therapies. ARDS associated with SARS-CoV-2 infection is characterized by clinical symptoms and laboratory findings that are consistent with a massive cytokine release syndrome, such as increased plasma levels of proinflammatory cytokines and altered lymphocyte subsets (2, 3). No new medication has been developed specifically for critical SARS-CoV-2 pneumonia (7–12), but based on the understanding of the pathophysiology of ARDS in COVID-19, several immunosuppressive strategies emerge as rational treatment approaches. Indeed, corticosteroids (13), Janus kinase (JAK) inhibitors that block cytokine signaling pathways such as Ruxolitinib (Ruxo) (14–21) or Baricitinib (22–25), the IL-6 antibody Tocilizumab (26–28) or the IL-1 receptor antagonist Anakinra (29, 30) were found to improve outcome in hospitalized COVID-19 patients. Intriguingly, the JAK1/2 inhibitor Baricitinib, which also targets the kinase AAK1, a regulator of endocytosis of the SARS-CoV-2 receptor ACE2, had been predicted as a promising treatment for COVID-19 by artificial intelligence algorithms as early as February 2020 (31).

At the University Hospital Marburg, following the successful individual treatment of a single patient (32), we conducted a non-randomized phase-II trial of the JAK1/2-Inhibitor Ruxolitinib in critically ill COVID-19 patients requiring mechanical ventilation (20). Ruxo was first approved for the treatment of myeloproliferative disorders (33), in which an activating mutation of JAK2 (V617F) is a common genomic finding (34). JAK2 is an intracellular tyrosine kinase that transduces signals from cytokine receptors, which in turn activate proliferative signaling cascades such as the MAP-kinase- or the PI3K/AKT-pathway (35). Beyond its antiproliferative impact on the cellular level, Ruxo also exerts immunosuppressive effects due to the integral function of JAK2 and its paralog JAK1 in cytokine networks, which are exploited clinically for the treatment of *graft-versus-host disease* (GvHD) following allogenic hematopoietic stem cell transplantation (36, 37). In this context, Ruxo not only acts via suppression of T-lymphocytes, but also of neutrophil granulocytes, which are major inducers of tissue damage in GvHD. In COVID-19, quantitative changes in neutrophils and monocytes have also been observed among patients with severe and moderate courses (38) as well as under treatment with Baricitinib (24). Moreover, inflammatory reactions in both GvHD and COVID-19 are at least partially mediated by the same cytokines, which include both proinflammatory mediators such as IL-6 or TNFα, and anti-inflammatory components such as IL-10 or TGFβ (39–41). In this study, we hypothesized that Ruxo’s mode of action in COVID-19-associated ARDS is reflected by changes in the peripheral blood proteome. To investigate this hypothesis, we applied mass spectrometry-based (MS) quantitative proteomics and cytometric bead array (CBA) analyses on serum samples from critically ill COVID-19 patients under treatment with Ruxo.

## 2. Materials and methods

### 2.1. Patients and samples

This study included 11 adult patients (age ≥ 18 years) with severe to critical COVID-19, who were treated at an Intensive Care Unit of the University Hospital Marburg between April 2020 and January 2022. All patients had not been vaccinated against SARS-CoV-2. SARS-CoV-2 infection was confirmed by polymerase chain reaction as described (20), yet, determination of SARS-CoV-2 variants was not included in the diagnostic routine. All patients were treated according to the current standard of care at the time of hospitalization. Eight patients were treated with Ruxo either on an individual basis or on a clinical trial (20). Informed consent to obtain and analyze samples for research purposes was obtained from all patients. Serum samples were stored at −80°C.

### 2.2. Serum proteomics

Samples were prepared for proteomic analysis by in gel digest (42, 43), as well as in solution digest (44) followed by high pH reversed phase separation (Pierce High pH Reversed-Phase Peptide Fractionation Kit, ThermoFisher Scientific) according to the manufacturer’s protocol, as reported. Briefly, after determining protein concentration of each serum sample by Lowry assay (BioRad Laboratories), 50 μg of total serum protein was separated into ten fractions using the in gel approach. For in solution digest, 150 μg were acetone-precipitated and separated into eight fractions. Liquid chromatography/tandem mass spectrometry was performed as reported (44). Used parameters were extracted and summarized using MARMoSET (45) and are included in the Supplementary Material 1. The mass spectrometry raw data from experiments described here has been deposited in the MassIVE member repository of the ProteomeXchange consortium (46).

### 2.3. Processing and statistical analysis of proteomics data

MS data were processed using MaxQuant v.2.0.3.0 (47), including label free quantitation against the human Uniprot protein sequence database (08.12.2020 download, canonical only with 75577 protein sequences).^1^ Parameters used for MaxQuant are included in the Supplementary Material 1. MaxQuant returned a file with 975 protein groups. 16 reverse proteins, 65 contaminant proteins and 194 proteins that were only represented by single peptides were dropped. The remaining 700 proteins were subjected to statistical analyses in R (48). For general linear model analysis we used the *autonomics* version 1.1.7.7 (49) interface fit_limma to the *limma* modeling engine (50). Overlap analyses of significantly regulated proteins identified in the *limma*-model was performed using the R-/Bioconductor package *VennDetail* (51). Functional analyses were performed using the R-/Bioconductor packages *clusterProfiler* (52) and *dbtORA* (53) and results were visualized using the package *enrichplot* (54).

### 2.4. Cytometric bead array assay

Fifty microliters of 1:4 diluted serum from each patient and time point was analyzed with human cytokine Grp I panel 17-plex cytometric bead array set (M5000031YV; Bio-Rad Laboratories), according to the manufacturer’s instructions and as described before (55) to quantify serum cytokines.

## 3. Results

### 3.1. Patient characteristics

This study included 11 COVID-19 patients, who were treated during the first to fourth wave of the pandemic (April 2020 - January 2022) at the University Hospital Marburg, Germany. All patients required intensive care treatment including mechanical ventilation (mean duration 26 days ± 10 days). Eight patients with ARDS were treated with Ruxo in a clinical trial (20) or on an individual basis (Ruxo*^only^* group), and two of these additionally received steroids (Ruxo^+*Steroids*^ subgroup) according to the standard of care at the time of hospitalization. One of three control patients (no ARDS, no Ruxo treatment) was treated with steroids. Baseline characteristics of the Ruxo and Control patients are summarized in Table 1. Blood samples for the analyses described in this work were collected before (day 0) and on days one (day 1), five to seven or nine to eleven days after initiation of Ruxo treatment. The latter time points were merged to day 6 and day 10, respectively, for statistical analyses. In the control group, different sampling time points are indicated relative to the day of ICU admission, which we considered the clinical peak of critical illness in these patients. All patients except for one in the Ruxo group who died on day 21, survived until day 28, which corresponds to the primary end point in several clinical trials investigating Ruxo in COVID-19-associated ARDS (20, 56) (Table 1).

**TABLE 1:** Characteristics of study patients.

	No./Median	Percentage (%) or range		
Total	11			
**Ruxolitinib**				
Yes	8	66.7%		
No	3	33.3%		
**Basic demographics**				
	**All patients n (%)** **median (range)**	**Ruxo patients n (%)** **median (range)**		**Control patients n (%)** **median (range)**
Female	4 (36.4%)	3/8 (37.5%)		1/3 (33%)
Male	7 (63.6%)	5/8 (62.5%)		2/3 (67%)
Age	65 (23–82)	61 (23–82)		70 (23–73)
BMI	27.7 (25.4–51)	29 (25.5–51)		27.7 (26.1–3.4)
**Comorbidities**				
	**All patients n (%)**	**Ruxo patients *n* (%)**		**Control patients n (%)**
Hypertension	8/11 (72.7%)	6/8 (75%)		2/3 (67%)
Obesity	6/11 (54.5%)	5/8 (62.5%)		1/3 (33%)
Cardiovascular (other than Hypertension)	4/11 (36.4%)	2/8 (25%)		2/3 (67%)
GIT diseases	4/11 (36.4%)	3/8 (37.5%)		1/3 (33%)
Diabetes	3/11 (27.3%)	2/8 (25%)		1/3 (33%)
Hyperlipidemia/Hyperlipoproteinemia	2/11 (18.2%)	1/8 (12.5%)		1/3 (33%)
CKD	2/11 (18.2%)	0/8 (0%)		2/3 (67%)
Malignancy	2/11 (18.2%)	1/8 (12.5%)		1/3 (33%)
Neurologic/Neuromuscular	1/11 (9.1%)	0/8 (0%)		1/3 (33%)
Thyroid	1/11 (9.1%)	1/8 (12.5%)		0/3 (0%)
**Previous medication**				
	**All patients n (%)**	**Ruxo patients n (%)**		**Control patients n (%)**
Betablockers	5/11 (45.5%)	4/8 (50%)		1/3 (33%)
PPIs	4/11 (36.4%)	2/8 (25%)		2/3 (67%)
ACE-inhibitors	3/11 (27.3%)	1/8 (12.5%)		2/3 (67%)
Antidiabetic medication	3/11 (27.3%)	2/8 (25%)		1/3 (33%)
Calcium antagonists	3/11 (27.3%)	2/8 (25%)		1/3 (33%)
Platelet aggregator inhibitors	3/11 (27.3%)	2/8 (25%)		1/3 (33%)
Statins	3/11 (27.3%)	1/8 (12.5%)		2/3 (67%)
Antineoplastic agents	2/11 (18.2%)	1/8 (12.5%)		1/3 (33%)
Immunosuppressive drugs (particularly corticosteroids, CNIs, rituximab)	2/11 (18.2%)	0/8 (0%)		2/3 (67%)
Diuretics	1/11 (9.1%)	1/8 (12.5%)		0/3 (0%)
NSAIDs and other analgesic drugs	1/11 (9.1%)	0/8 (0%)		1/3 (33%)
Psychoactive drugs	1/11 (9.1%)	1/8 (12.5%)		0/3 (0%)
Thyroid medications	1/11 (9.1%)	1/8 (12.5%)		0/3 (0%)
**Outcome**				
Days of hospitalization – median (range)	29 (21–67)	28.5 (21–65)		40 (23–67)
Alive at day 28	10/11 (91%)	7/8 (87.5%)		3/3 (100%)
Discharged	7/11 (63.6%)	5/8 (62.5%)		2/3 (67%)
Deceased	4/11 (36.4%)	3/8 (37.5%)		1/3 (33%)

ACE, angiotensin-converting enzyme; BMI, body mass index; CKD, chronic kidney disease; CNI, calcineurin inhibitors; GIT, gastro-intestinal tract; NSAIDs, non-steroidal anti-inflammatory drugs; PPIs, proton-pump inhibitors.

### 3.2. Serum proteomes of critically ill COVID-19 patients with or without Ruxo-treatment

**FIGURE 1:**
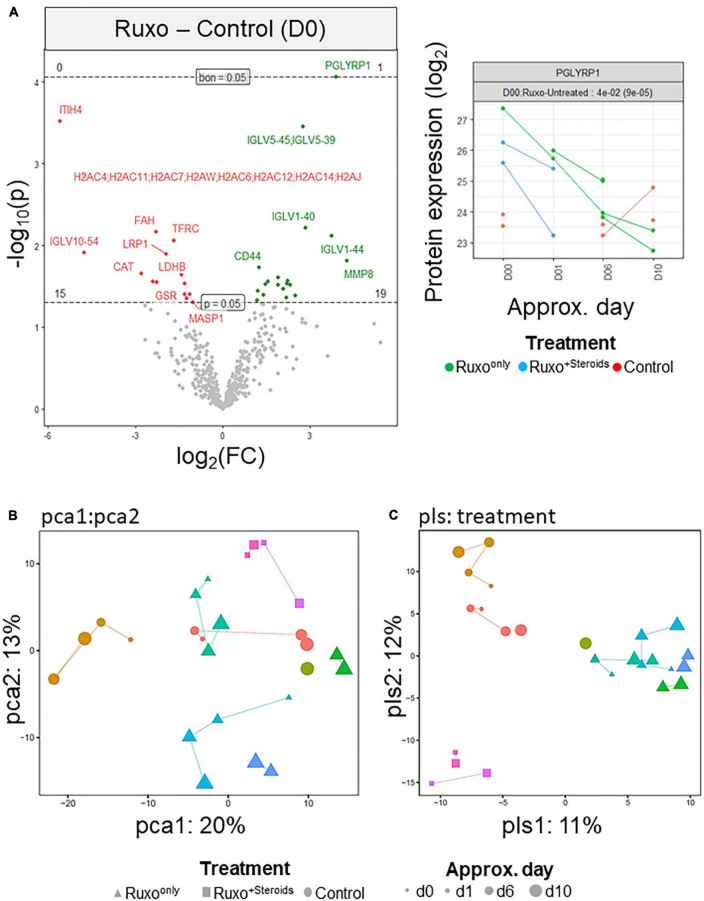
Serum proteomes of critically ill COVID-19 patients with and without Ruxo treatment. **(A)** Left panel: Volcano plot of MS data indicating differential protein expression between COVID-19 patients with or without ARDS [Ruxo group (green) vs. control group (red)]. “p” indicates the raw *p*-value, “bon” indicates the Bonferroni-corrected *p*-value. Right panel: Time trajectory from principal component analysis for the protein PGLYRP1. **(B)** Principal component analysis (PCA) score plot derived from mass spectrometry (MS) data of different patients and sampling time points using treatment as a design-factor. Each individual is color-coded. Additionally, for each subject, “Treatment” is coded by symbol shape and “ApproxDay” by size. **(C)** Partial least square regression analysis (PLS) derived from the MS data. Coding as in panel **(B)**.

To explore the serum proteomes of patients with severe COVID-19-associated pneumonia or ARDS and the impact of Ruxo in the latter condition, we performed MS in the absence of any depletion protocol against high-abundant serum proteins on serum samples collected at different time points after initiation of treatment. In total 25 samples from nine patients were investigated including three control, four Ruxo*^only^* and two Ruxo^+*Steroids*^ patients. We observed differential protein expression between Ruxo-treated and untreated patients at day 0 with Peptidoglycan recognition protein 1 (PGLYRP1 as the most significant upregulated factor in the treatment group (Figure 1A). However, principal component analysis (PCA) revealed clear patient-specific effects and time trajectories, which, did not generalize across patients. Subtle treatment effects were only recognizable for the Ruxo^+*Steroids*^ subgroup (Figure 1B). Thus, the main PCA drivers appeared to be factors unrelated to the study-design. Particularly in the control group, covariates associated with preexisting conditions and/or patients’ permanent medications such as chronic kidney disease, diabetes, immunosuppression or antihypertensive drugs, confirmed, that the largest variability in the dataset was not caused by different treatments for COVID-19 (Supplementary Figure 1A in Supplementary Material 3). To further investigate potential effects of our experimental design, we performed partial least square (PLS) regression analysis. In line with the PCA results, examination of “treatment” as a design-factor revealed no clear separation of groups, (Figure 1C). These calculations underlined considerable heterogeneity of individual patients in all treatment groups in our limited dataset.

### 3.3. Changes in serum proteomes of COVID-19 patients under Ruxo treatment over time

**FIGURE 2:**
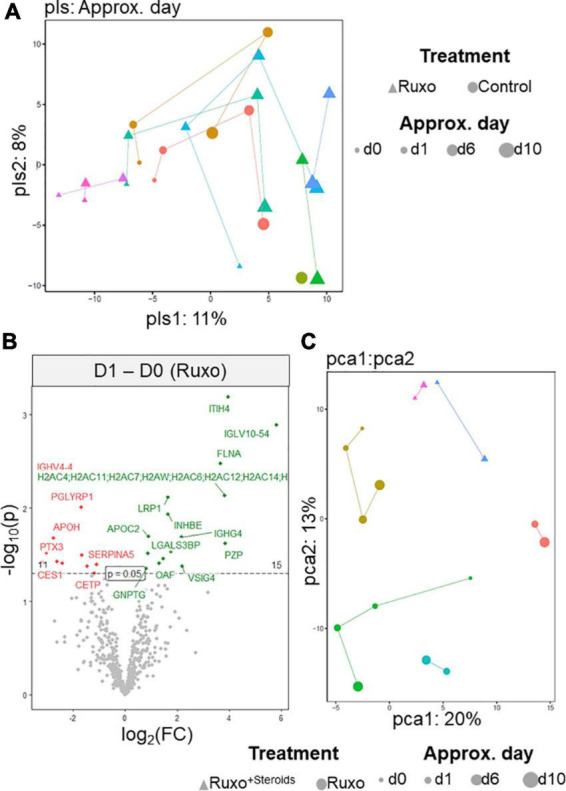
Changes in serum proteomes of COVID-19 patients upon Ruxo treatment over time. **(A)** Partial least square regression analysis (PLS) derived from the MS data of different patients and sampling time points using time as a design-factor. Each individual is color-coded. Additionally, for each subject, “Treatment” is coded by symbol shape and “ApproxDay” by size. Samples from the same subject are connected by a line. **(B)** Volcano plot of MS data indicating differential protein expression in COVID-19 patients under treatment with Ruxo at day 1 (green) compared to day 0 (red). “p” indicates the raw *p*-value, “bon” indicates the Bonferroni-corrected *p*-value. **(C)** General linear modeling of protein expression as a function of sampling day.

Given that “treatment” did not allow to distinguish patients treated with Ruxo from untreated patients, we next investigated “time” as a relevant design-factor in our experimental setting by PLS. Indeed, we detected some generalizable time effects, which were most pronounced (i.e., displayed the highest PLS1 loadings) for the proteins Afamin (AFM), Apolipoprotein C3 (APOC3), Lipopolysaccharide binding protein (LBP), and Serpin family A member 5 (SERPINA5) (Figure 2A and Supplementary Figure 1B in Supplementary Material 3). Yet, these exploratory analyses still revealed considerable heterogeneity within the three non-Ruxo patients, precluding to use them as an appropriate control group. Thus, these patients were excluded from subsequent analyses.

Considering only Ruxo-treated patients (Ruxo*^only^* and Ruxo^+*Steroids*^), we next investigated differential protein expression at different time points. Inter-Alpha-Trypsin Inhibitor Heavy Chain 4 (ITH4) was most significantly upregulated while, PGLYRP1 was expressed at lower levels under treatment (Figure 2B). This latter observation was consistent with our initial finding that PGLYRP1 was less abundant in the less severely ill patients. Subsequently, we extended our time-course analyses and performed general linear modeling of protein expression as a function of collection day, including subject as a random effect. This analysis revealed four proteins which changed systematically (FDR < 0.05) across patients, although these were not measured in all patients and/or at all time points: Apolipoprotein A5 (APOA5), N-Acetylglucosamine-1-phosphate transferase subunit gamma (GNPTG), PGLYRP1 and Serpin family A member 1 (SERPINA1) (Figure 2C, Supplementary Figure 1C in Supplementary Material 3, and Supplementary Table 1 in Supplementary Material 2). Modifying this analysis by calculating Helmert contrasts, i.e., comparing each day against the average of the previous time points confirmed systematic time-effects on APOA5 and SERPINA1 and added one more protein, Immunoglobulin Heavy Variable 3/OR16-12 (IGHV3OR16-12), a poorly characterized immunoglobulin complex component (Supplementary Figure 1C in Supplementary Material 3). The statistically significant time-effect observed for SERPINA1 attracted our particular attention, as this protein was detected in the two Ruxo^+*Steroids*^ patients at d0 and d1 at approximately identical levels, but appeared completely absent at later time points. Moreover, when we compared the proteomes of critically ill COVID-19 patients who eventually deceased due to the infection to those who could be discharged from hospital, we found SERPINA1 among the factors that were higher expressed in the survivors. This observation was also remarkable because most of the factors that differentiated between final outcomes were largely unchanged over time (Supplementary Figure 2 in Supplementary Material 3). Due to the heterogeneity of our dataset, we could not confirm the time-effect of SERPINA1 as a true switch-like response, as a global analysis of presence/absence schematics in our dataset exhibited a random pattern (data not shown). In addition, since we had also collected peripheral blood mononuclear cells (PBMCs) from some patients at day 0, day 1 and day 6, we applied qPCR to examine whether changes in serum protein levels were paralleled by mRNA expression changes in these cells. However, this seemed not to be the case, which we attribute to differential expression of candidate genes (GNPTG, HP, C4B, PGLYRP1, WARS1, and SERPINA1) in various PBMC-subsets as well as expression in other tissues which contribute to serum levels of these proteins, such as liver (data not shown). Taken together, these results suggested that a mode of action for Ruxo in a small cohort of critically ill COVID-19 patients is potentially more reliably deduced from longitudinal in-patient effects rather than from comparisons between treatment groups.

### 3.4. Functional analysis of differentially regulated proteins under Ruxo treatment compared to baseline

**FIGURE 3:**
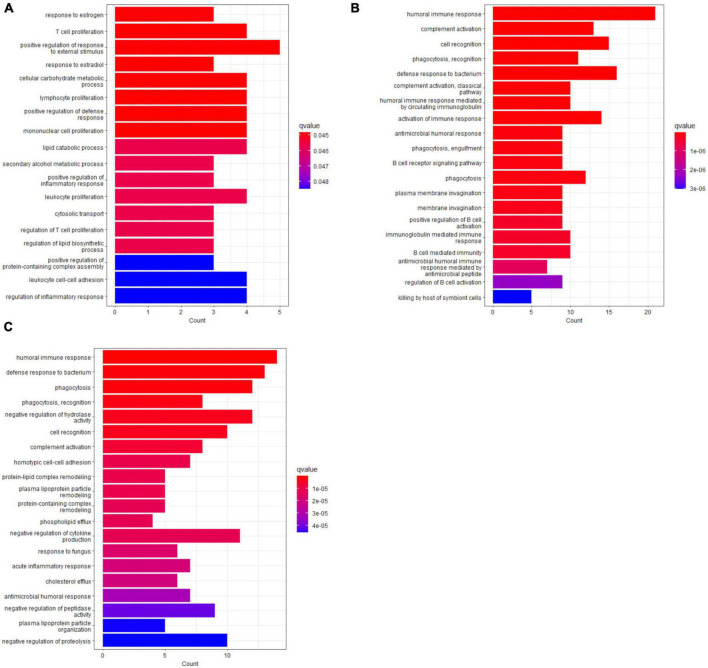
Overrepresentation analysis of differentially regulated serum proteins in COVID-19 patients under Ruxo treatment. ORA was performed on differentially regulated proteins (raw *p*-value < 0.05) as detected by MS on **(A)** day 1, **(B)** day 6, and **(C)** day 10. The top 20 GO terms of the category biological process from analyses using the *clusterProfiler* package were plotted. Note that direction of regulation (up or down) was not considered in this analysis. The barplots indicate the level of significance and the number of included genes for each term. See Supplementary Tables 6–8 in Supplementary Material 2 for complete ORA results.

**FIGURE 4:**
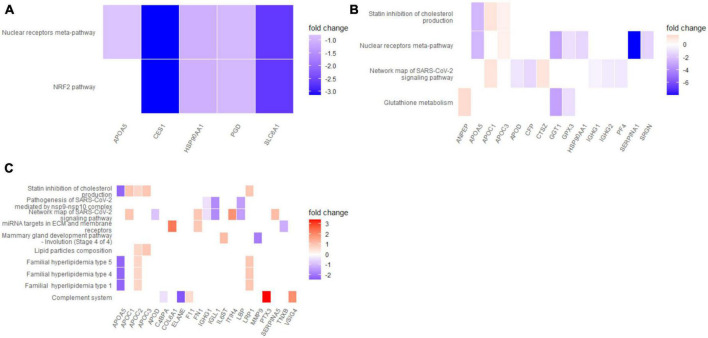
Pathway enrichment analysis of differentially regulated serum proteins in COVID-19 patients under Ruxo treatment. Wiki-Pathway enrichment analysis was performed on differentially regulated proteins (raw *p*-value < 0.05) as detected by MS on **(A)** day 1, **(B)** day 6 and **(C)** day 10. Heatmap-like plots indicate expression of individual genes involved in each pathway.

To further characterize the response to Ruxo in COVID-19-ARDS patients on a functional level, we performed gene ontology (GO) and pathway enrichment analyses on the proteins that were differentially regulated at different time points according to our linear model. Each treatment day was compared to day 0 separately since the time trajectories from PLS analyses indicated opposite effects on several proteins over time. Focusing on significant proteins (raw *p*-value < 0.05) we identified ten proteins that were upregulated upon Ruxo treatment at day 1 and 17 proteins that were downregulated. At day 6 and 10, 22 and 47, or, respectively, 32 and 40 proteins were up- or downregulated (Supplementary Tables 2–4 in Supplementary Material 2). Overlap analyses of affected proteins at day 1, 6, and 10 confirmed opposite regulation of several factors as indicated by PLS. We identified only five factors that were regulated both concordantly and significantly over time (up: IGLV10-54, PSMB1, down: PGLYRP1, APOA5, WARS1, Supplementary Table 5 in Supplementary Material 2). Overrepresentation analysis (ORA) of GO terms (52) including all significant proteins (raw *p*-value < 0.05) at any individual time point revealed enrichment of biological processes that implicated a T-cell response only on day 1, but not on days 6 and 10 (Figure 3 and Supplementary Tables 6–8 in Supplementary Material 2). The highest fold change of T-cell-proliferation-related proteins was observed for VSIG4, a negative regulator of this process (57), which was upregulated approximately 2-fold (Supplementary Figure 3 in Supplementary Material 3). On the later time points, we found significant enrichment of the humoral immune response with a marked focus on B-cell-dependent processes on day 6, as well as complement activation. Notably, most proteins relating to these terms were downregulated (Figures 3B, C and Supplementary Tables 6–8 in Supplementary Material 2). Similar results were obtained applying a different implementation of ORA (dbtORA (53), Supplementary Tables 9–11 in Supplementary Material 2). Pathway enrichment analysis using the curated Wiki pathway database (52) yielded only two gene sets, the *Nuclear receptors meta-pathway* and the *NRF2-pathway* at day 1. The first one was also enriched on day 6, together with additional pathways including *Network map of SARS-CoV-2 signaling* and *Statin inhibition of cholesterol production*. These SARS-CoV-2- and Cholesterol-gene sets in turn were shared by enrichment results for day 6 and day 10 (Figure 4). Of note, most of the affected pathways included APOA5, which was downregulated under Ruxo treatment at all time points. Although enrichment analyses are less robust for the day 1 time point due to a very short list of only 27 significantly regulated proteins (compared to 69 on day 6 and 72 on day 10), these results suggest that Ruxo exerts immediate, but transient effects in COVID-19-ARDS patients, that during the course of several days clearly connect to the underlying condition, namely SARS-CoV-2-infection.

### 3.5. Serum cytokine levels in COVID-19-ARDS and effects of Ruxo treatment

**FIGURE 5:**
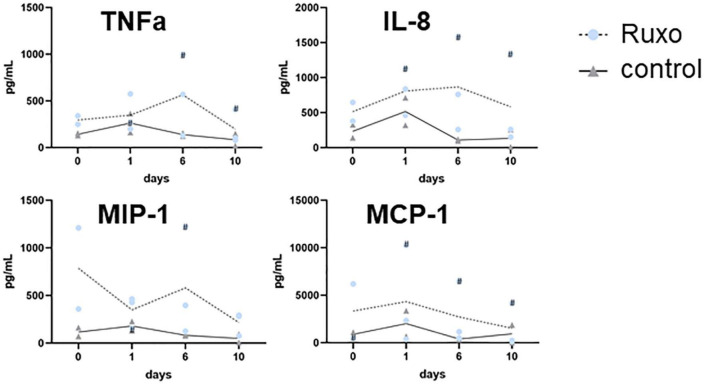
Serum cytokine levels in treated and untreated COVID-19 patients. Cytometric bead array assay performed with serum samples collected at different time points (day 0, day 1, day 6, and day 10) from three COVID-19 patients under Ruxo treatment and two control patients, without Ruxo treatment. One patient in the Ruxo group also received steroids (marked by #).

Cytokines and chemokines are generally difficult to capture by MS because of very low serum concentrations compared to other serum proteins. We therefore investigated these mediators in COVID-19-ARDS compared to COVID-19-pneumonia and their potential regulation by Ruxo separately using a cytometric bead array (CBA) assay. This analysis was restricted to three Ruxo and two control patients from whom sufficient sample material was available. COVID-19 patients with ARDS exhibited higher serum levels of all cytokines and chemokines measured (IFNy, TNFa, IL-4, IL-6, IL-7, IL-8, IL-10, IL-13, MIP-1b, and MCP-1) compared to patients with COVID-19 pneumonia without ARDS (data not shown). Moreover, serum cytokines and chemokines clearly showed patient-specific time courses, as observed in our proteomics experiments. However, despite heterogenous time-patterns, several mediators in Ruxo patients tended to approximate control levels after 10 days of treatment, such as TNFa, IL-8, MIP-1b, and MCP-1 (Figure 5). This observation presumably reflects attenuation of the cytokine storm, consistent with the expected clinical effects of Ruxo, although with slower kinetics than expected based on our initial clinical experience with Ruxo (32) and even transient increase of proinflammatory cytokines (58).

## 4. Discussion

Ruxo has been repurposed for the treatment of SARS-CoV-2 infection in different clinical settings inside and outside of clinical trials, but the clinical significance of this drug in COVID-19 pneumonia and ARDS remains to be firmly established (18, 20, 21, 32, 56, 59–62). The work presented here adds to previous work on the mechanisms of action of Ruxo in hyperinflammation and respiratory distress (63, 64). Specifically, we aimed to gain deeper insights into systemic effects of Ruxo in critical COVID-19 by studying serum proteomes by MS and cytokine array analyses at different time points after initiation of treatment. Based on our early clinical experience with Ruxo for ARDS associated with SARS-CoV-2 infection (32), we expected rapid and profound changes of circulating factors. We therefore analyzed samples from only eight COVID-19-ARDS patients treated with Ruxo and three controls and did not stratify the patients/samples investigated here for outcome.

Firstly, we found time trajectories in the proteomics data that generalized for all patients, which included factors that have been mentioned in the literature in the context of COVID-19 such Afamin (65), APOC3 (66), or SERPINA5 (67). On the other hand, time patterns for a set of different proteins including APOA5, GNPTG or PGLYRP1 only became detectable after excluding the extremely heterogenous control group from further analyses. Only 5 factors were regulated both concordantly and significantly over time, including Immunoglobulin Lambda Variable 10–54 (IGLV10-54) and Proteasome 20S Subunit Beta 1 (PSMB1), which were upregulated and, respectively, PGLYRP1, APOA5 and Tryptophanyl-tRNA Synthetase 1 (WARS1), which were downregulated. IGLV10-54 has been identified as one of the top upregulated genes in SARS-CoV-2 infected individuals compared to healthy controls and also as component of an immune-response related gene cluster that distinguishes Long-COVID-patients from individuals who had recovered from the disease (68, 69). PSMB1, along with other proteasomal subunits has been described to be induced by hypoxia in the context of SARS-CoV-2-infection (70). In addition, certain PGLYRP1-derived peptides have been described to inhibit proinflammatory cytokine-production in a mouse model of acute lung injury with diffuse alveolar damage (71). We have not examined individual peptides on a sub-protein level in our analysis, but in view of these previous results, decrease of PGLYRP1 under Ruxo treatment might not necessarily point out PGLYRP1 as a direct target of Ruxo, but rather indicate resolution of the ARDS-causing cytokine storm within several days. APOA5 has been described to be differentially regulated in severe COVID-19 compared to healthy controls and also during recovery from this condition (72). Finally, WARS1, which has been reported to boost the innate immune response as a ligand of toll-like receptors TLR2 and TLR4 (73), has been identified as a factor involved in several biological processes associated with COVID-19 severity and has been described to be downregulated on the mRNA-level upon SARS-CoV-2-infection (74, 75).

On the functional level, i.e., with regard to biological processes or cellular pathways we found two phases of the response to Ruxo. The early phase on day 1 following treatment initiation was characterized by a T-cell response and repression of the NRF2-pathway, reflecting well established actions of Ruxo as a mediator of T-cells (76) and a previously identified SARS-Cov-2 key pathogenic pathway (77). At later time points, however, we observed enrichment of other SARS-CoV-2-related pathways, which involved, for example, ITIH4. This protein, which acts as a protease inhibitor upon proteolytic cleavage (78), has been detected at increased levels in plasma or serum samples of COVID-19 patients in previous proteomics studies reported in the literature (79, 80) and has also been proposed as a potential predictor for disease mortality (81). These observations support the clinical experience that Ruxo exerts prompt effects in COVID-19-associated ARDS, which only transiently overlay more sustained immune responses or pathomechanisms.

Thus, our careful and detailed analyses of our dataset revealed several lines of evidence that the mechanism of action of Ruxo in COVID-19-ARDS can be related to both known effects of this drug and the clinical condition studied, i.e., SARS-CoV-2-infection. However, interpretation of our experiments is clearly compromised by the very limited number of Ruxo- and control patients that were included in this study, which resulted in extensive variability within our cohort with regard to clinical covariates, and thus, of our proteomics dataset. Moreover, given that we included patients with critical COVID-19 from the first to fourth wave of the pandemic, variant-specific proinflammatory effects of different SARS-CoV-2-mutants may also have contributed to the heterogeneity observed in our dataset (82).

In summary, the results presented here further strengthen the concept of Ruxo constituting a rational treatment for COVID-19-related ARDS that warrants further preclinical and clinical investigation.

## Data availability statement

The mass spectrometry proteomics data presented in this study have been deposited to the ProteomeXchange Consortium (http://proteomecentral.proteomexchange.org) via the MassIVE partner repository with the data set identifier PXD041909 and http://doi.org/10.25345/C5000094G.

## Ethics statement

The studies involving human participants were reviewed and approved by the Clinical Ethics Committee of the Faculty of Medicine, Philipps-University Marburg (No. 57/20). The patients/participants provided their written informed consent to participate in this study.

## Author contributions

AN, HR, JG, CS, and EM conceptualized the study. TT, IK, TW, BB, JH, CK, and AB contributed to the sample acquisition. SV, TT, LS, and HM designed and performed the experiments. AMB, JG, SV, and EM analyzed the mass spectrometry data. SV and EM wrote the first draft of the manuscript with contributions from AMB and TT. All authors contributed to the manuscript revision, read, and approved the submitted version.

## Footnotes

1
https://www.uniprot.org/
