# Supplementary material for: Serum proteomics hint at an early T-cell response and modulation of SARS-CoV-2-related pathogenic pathways in COVID-19-ARDS treated with Ruxolitinib
Source: Front Med (Lausanne). 2023 May 24;10:1176427. doi: 10.3389/fmed.2023.1176427 (PMC10244732; doi:10.3389/fmed.2023.1176427)
Supplement: Supplementary file 1 [file Data_Sheet_1.zip › Supplementary file 3_proof.docx]

**Serum proteomics hint at an early T-cell response and modulation of SARS-CoV-2-related pathogenic pathways in COVID-19-ARDS treated with Ruxolitinib**

Sara Völkel, Thomas S. Tarawneh, Laura Sacher, Aditya M. Bhagwat, Ihab Karim, Hildegard I.D. Mack, Thomas Wiesmann, Björn Beutel, Joachim Hoyer, Christian Keller, Harald Renz, Andreas Burchert, Andreas Neubauer, Johannes Graumann, Chrysanthi Skevaki, Elisabeth K.M. Mack

**Supplementary figures and figure legends**

**
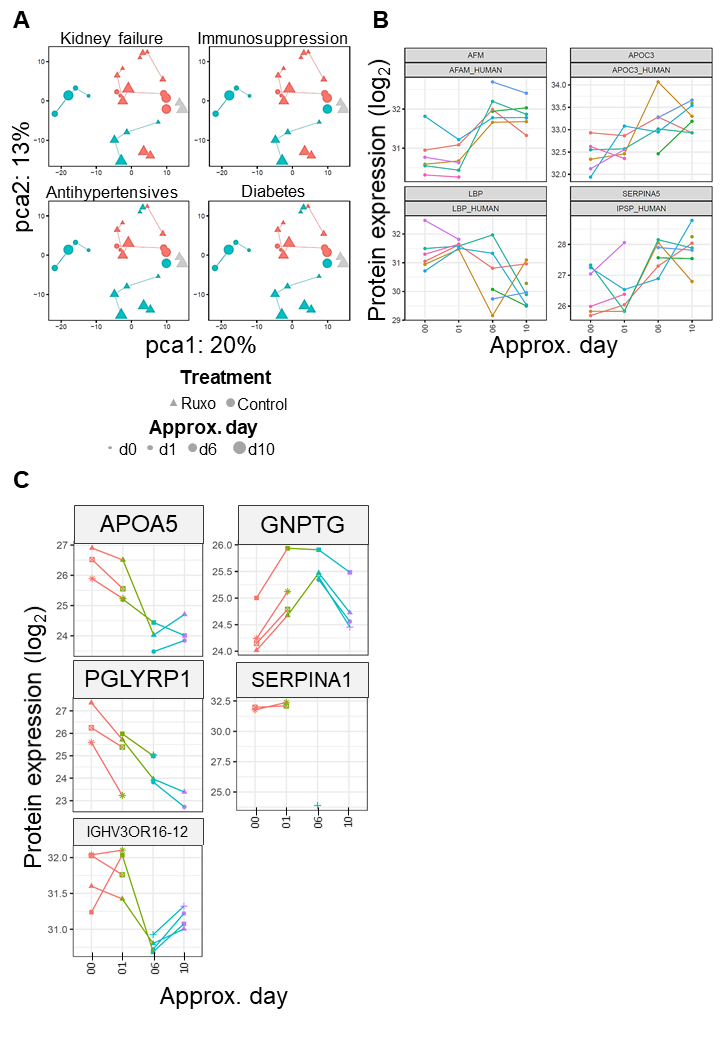
**

**Figure S1. Serum proteomes of critically ill COVID-19 patients with and without Ruxo treatment.** (A) Principal component analysis (PCA) score plot derived from mass spectrometry (MS) data of different patients and sampling time points using treatment as a design-factor and comorbidities or patients’ permanent medications as covariates. For each subject, ‘Treatment’ is coded by symbol shape and ‘ApproxDay’ by size, and the indicated covariates are color-coded (red = absence of condition, blue = presence of condition). (B) Partial least square regression analysis (PLS) derived from the MS data of different patients and sampling time points using time as a design-factor. Time trajectories for the proteins with the highest PLS1 loadings are indicated. Individuals are color-coded and samples from the same subject are connected by a line. (C) General linear modeling of protein expression as a function of sampling day. The indicated proteins changed systematically accross patients (FDR < 0.05) over time.


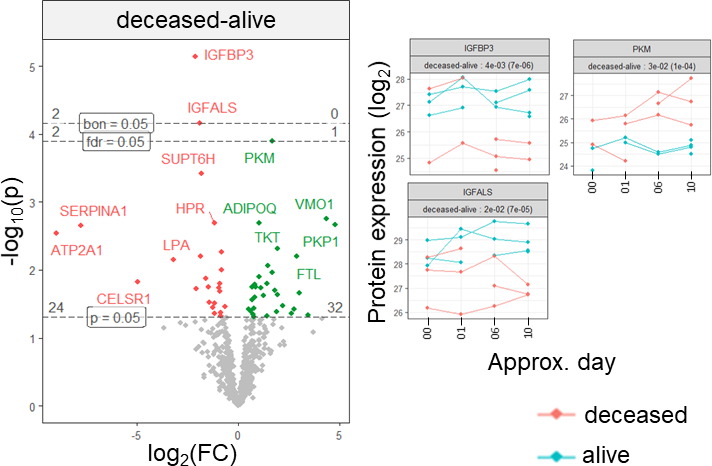


**Figure S2. Differential expression analysis of serum proteomes in COVID-19 patients according to outcome.** Left panel: Volcano plot of MS data indicating differential protein expression between COVID-19 patients who eventually deceased due to the infection (green) and patients who could be discharged from hospital alive (red). All patients and all time points were included in the analysis. ‘p’ indicates the raw p-value, ‘fdr’ the FDR-corrected p-value and ‘bon’ the Bonferroni-corrected p-value. Right panels: Time trajectories for the proteins that were significantly differentially expressed between deceased and alive patients from general linear modeling of protein expression as a function of sampling day. Samples from the same subject are connected by a line.


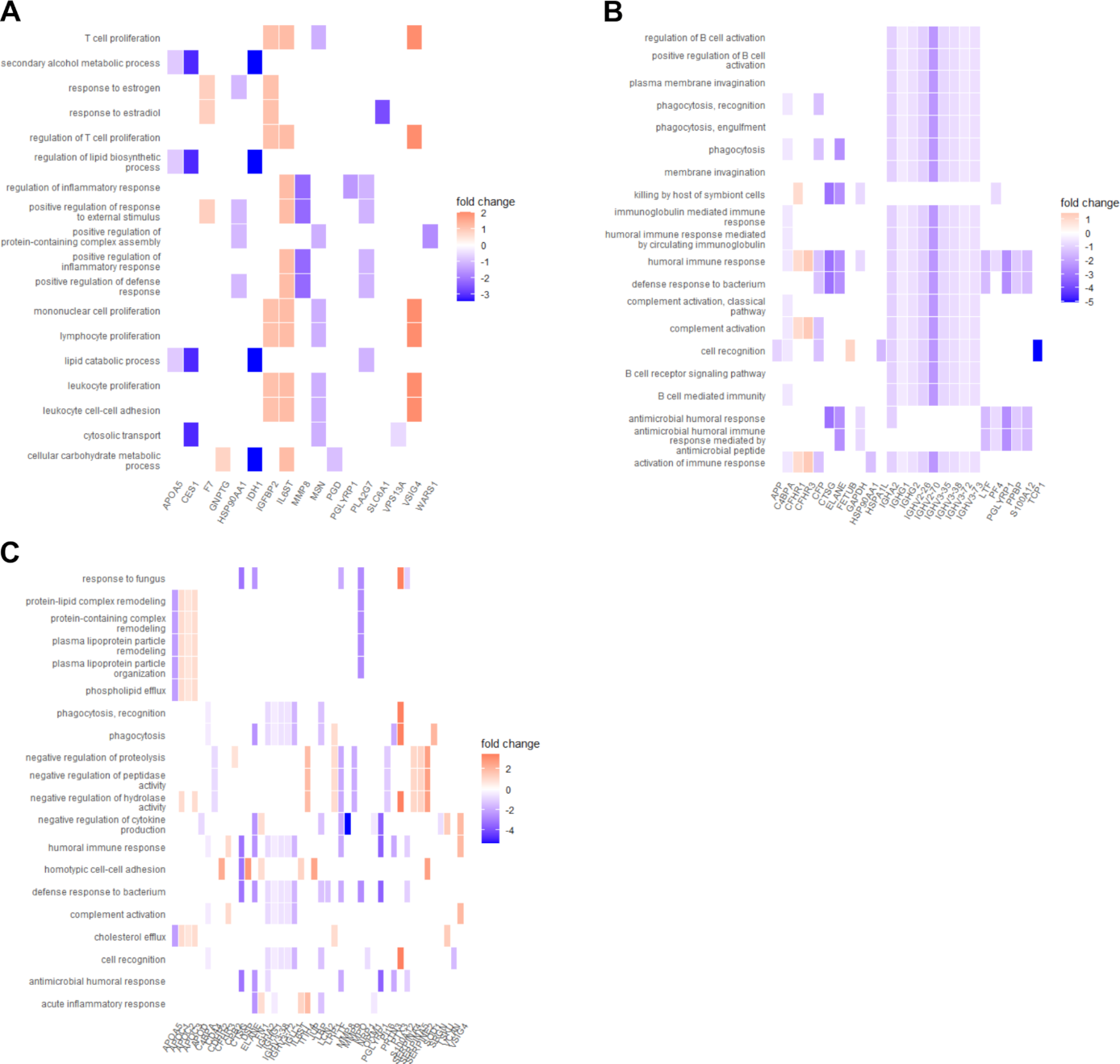


**Figure S3. Overrepresentation analysis of differentially regulated serum proteins in COVID-19 patients under Ruxo treatment.** ORA was performed on differentially regulated proteins (raw p-value < 0.05) as detected by MS on (A) day 1, (B) day 6 and (C) day 10. The top 20 GO terms of the category biological process from analyses using the clusterProfiler package were plotted. The heatmap-like plots indicate expression of individual genes included in each term. See supplementary tables S6-S8 for complete ORA results.
